# Protein-bound uremic toxins as therapeutic targets for cardiovascular, kidney, and metabolic disorders

**DOI:** 10.3389/fendo.2025.1500336

**Published:** 2025-01-27

**Authors:** Shihan Zhang, Shasha Tang, Yalei Liu, Binghua Xue, Qinyuan Xie, Lingyun Zhao, Huijuan Yuan

**Affiliations:** Department of Endocrinology, Zhengzhou University People’s Hospital, Henan Provincial People’s Hospital, Henan Provincial Key Medicine Laboratory of Intestinal Microecology and Diabetes, Zhengzhou, China

**Keywords:** protein-bound uremic toxins, gut microbiota, cardiovascular-kidney-metabolic syndrome, metabolic diseases, management

## Abstract

Cardiovascular-kidney-metabolic (CKM) syndrome is a systemic clinical condition characterized by pathological and physiological interactions among metabolic abnormalities, chronic kidney disease, and cardiovascular diseases, leading to multi-organ dysfunction and a higher incidence of cardiovascular endpoints. Traditional approaches to managing CKM syndrome risk are inadequate in these patients, necessitating strategies targeting specific CKM syndrome risk factors. Increasing evidence suggests that addressing uremic toxins and/or pathways induced by uremic toxins may reduce CKM syndrome risk and treat the disease. This review explores the interactions among heart, kidney, and metabolic pathways in the context of uremic toxins and underscores the significant role of uremic toxins as potential therapeutic targets in the pathophysiology of these diseases. Strategies aimed at regulating these uremic toxins offer potential avenues for reversing and managing CKM syndrome, providing new insights for its clinical diagnosis and treatment.

## Background

In recent years, chronic kidney disease (CKD) has become a global epidemic, with a prevalence rate as high as 14.3% (1). The incidence of CKD is increasing due to rising rates of diabetes, hypertension, and obesity. In China, the adult prevalence of CKD is 10.6% (2). Its complex pathogenesis and lack of effective interventions lead to multisystem complications (3), making it a significant global public health concern. CKD-related diseases caused approximately 1.2 million deaths worldwide in 2017, with this number projected to increase to 2.2 million by 2040 (4). Cardiovascular disease (CVD) is the most common complication and leading cause of death associated with CKD (5). Metabolic, cardiovascular, and kidney diseases significantly overlap, presenting major health challenges with high morbidity and mortality rates. These conditions often coexist and interact, underscoring the interconnectedness of heart, kidney, and metabolic health. In 2023, the American Heart Association (AHA) proposed a new disease concept—Cardiovascular-Kidney-Metabolic (CKM) syndrome, emphasizing the pathophysiological interactions among metabolic risk factors, CKD, and CVD. This syndrome is characterized by systemic diseases leading to multi-organ dysfunction and increased cardiovascular adverse events (6).

A long-term retrospective study in Sweden, involving over a million patients with diabetes mellitus (DM) and control subjects, with a median follow-up of 7.5 years, observed an increasing trend in end-stage renal disease (ESRD) among DM patients compared to controls. The incidence rate of ESRD (per 100,000 person-years) was 116.1 in patients with DM versus 42.0 in the control group. Further analysis revealed associations between cardiac metabolic risk factors in patients with DM and increased risk of ESRD, including elevated glycated hemoglobin, systolic blood pressure, body mass index, advanced age, and low high-density lipoprotein cholesterol levels (7).Studies have shown that 93.6% of patients with type 2 diabetes mellitus (T2DM) have at least one concurrent cardiovascular-kidney-metabolic disease, with 51% having three or more (8). Epidemiological research and clinical trial data indicate that successfully controlling multiple CVD risk factors can reduce the risk of CVD events by ≥50% (9).

Increasing evidence suggests that the progression of metabolic, cardiovascular, and kidney diseases is associated with the accumulation of uremic toxins, particularly in CKM syndrome stages II-IV (10). Elevated levels of indoxyl sulfate (IS), were found in urine samples from patients with DM and correlated with changes in proteinuria (11, 12). A 5-year follow-up study involving 521 patients with CKD confirmed that impaired kidney function and increased uremic toxins accelerate atherosclerosis. Higher plasma levels of colonic uremic toxins such as trimethylamine-N-oxide (TMAO) in CKD subjects were associated with a 2.8-fold increased risk of CVD-related mortality, and worsened overall survival with increasing TMAO levels (13). In animal experiments, administering a TMAO inhibitor to mice on a high-choline diet reduced plasma TMAO levels and foam cell formation, thereby improving atherosclerotic plaque formation, suggesting a potential therapeutic approach for treating cardiac metabolic diseases (14, 15). Clinical management of CKM syndrome currently focuses on stage-specific drug selection and multidisciplinary approaches. Although conventional treatments, such as lifestyle interventions, glycemic control, and pharmacotherapy, can delay the progression of CKM syndrome, the incidence of adverse heart-kidney outcomes remains high. Patients with both DM and kidney disease have an all-cause mortality rate approximately 30 times higher than those with diabetes alone (16). Even with comprehensive treatment strategies, disease progression is not fully prevented, with a 41.6% mortality rate over a 14-year follow-up (17). Large-scale cohort studies have confirmed that baseline CKD, CVD, and cardiac-kidney comorbidity significantly increase the risk of all-cause mortality by 1.5-fold, 1.8-fold, and 2.4-fold, respectively, compared to those without these conditions. Thus, there is an urgent need to explore therapeutic strategies that reduce the incidence and mortality of CKM syndrome. Uremic toxins such as IS and TMAO have emerged as specific risk factors. Strategies aimed at regulating these uremic toxins offer potential avenues for reversing and managing CKM syndrome, providing new insights for its clinical diagnosis and treatment.

## Unique environment of protein-bound uremic toxins in CKM syndrome

### Production and metabolism of gut-derived uremic toxins

Currently, over 100 uremic toxins have been identified in the serum of patients with CKD, including a type known for its high protein affinity, called protein-bound uremic toxins (PBUTs). PBUTs, such as IS, p-cresyl sulfate (PCS), and TMAO, primarily originate from endogenous metabolic products, intestinal bacterial breakdown, and exogenous intake. As renal failure progresses, dysbiosis of the gut microbiota, changes in enzymatic activity, and ineffective renal excretion lead to the accumulation of PBUTs, exacerbating multi-organ damage, including to the heart and kidneys (18–20).

Many uremic toxins derive from dietary components such as tryptophan, tyrosine, and choline metabolism. Gut bacteria metabolize dietary tryptophan into uremic toxin precursors, which pass through the intestinal mucosal barrier via the portal vein to the liver. There, they couple with sulfate ions to transform into PBUTs, IS (21). Tyrosine and phenylalanine metabolites generate p-cresol, which is absorbed by the intestines and converted to PCS through the action of sulfotransferases in intestinal epithelial cells, with a small portion metabolized in the liver into glucuronide-conjugated p-cresol (22). Phenylacetic acid in host liver cells forms phenylacetylglutamine (PAGln) (23). Trimethylamine (TMA) is absorbed and circulates to the liver, where it is converted to TMAO (24). Ultimately, uremic toxin precursors are transformed into PBUTs and secreted via renal tubules into urine (25).

**Figure 1:**
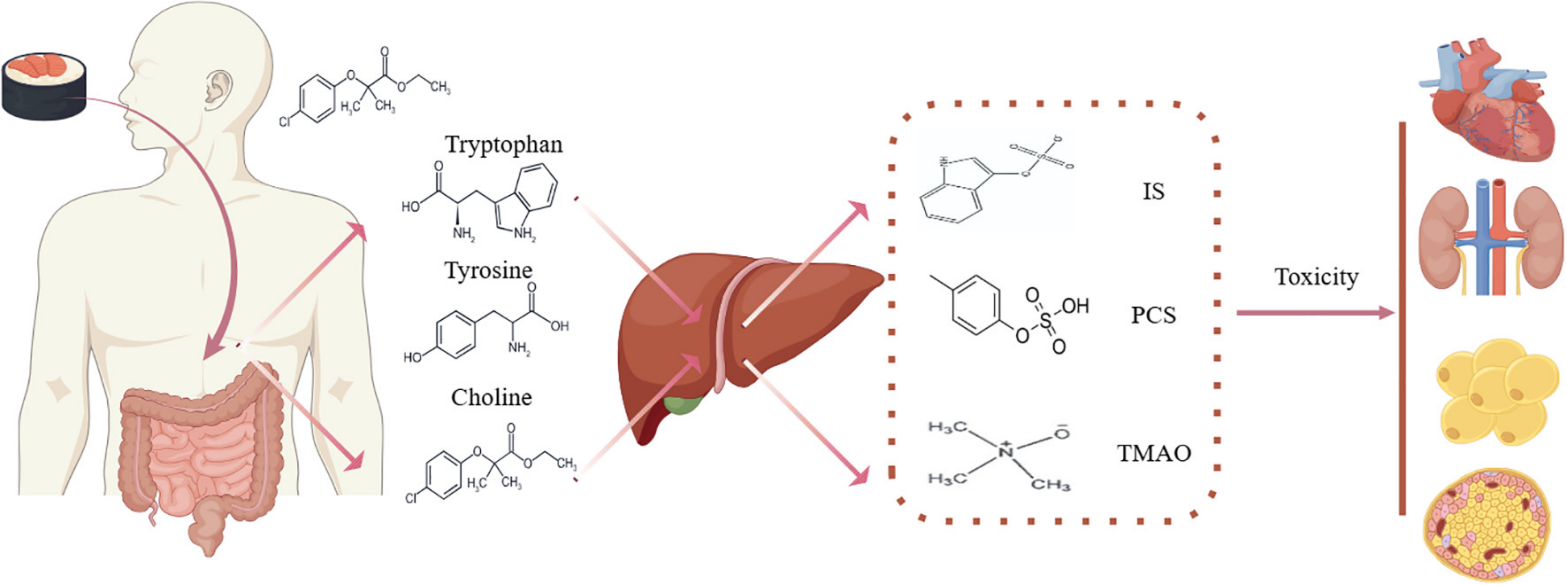
Protein-bound uremic toxins in CKM syndrome. IS, indoxyl sulfate; PCS, p-cresol; TMAO, trimethylamine-N-oxide.

In renal tissue, organic anion transporters (OATs) on the basolateral side of proximal tubule cells play a crucial role in the selective absorption of PBUTs from the blood and their active secretion into the renal tubular lumen (26, 27). In patients with CKD, the abnormal increase in uremic toxins primarily stems from changes in intestinal microbiota, leading to increased toxin precursors and reduced secretion through renal tubules due to declining kidney function. In the context of PBUTs accumulation in the bloodstream, increased OATs expression stimulated by CKD leads to significant PBUTs secretion. However, further renal tubular toxicity and fibrosis worsen renal function, failing to compensate for toxin accumulation. Accumulated toxins can specifically bind to the aryl hydrocarbon receptor (AhR), participating in systemic intracellular signaling pathways related to uremic toxicity, and contributing to the occurrence of CKD and its complications, such as CVD (28) (**Figure 1**).

### Interaction between PBUTs and CKD

According to the “gut-kidney axis” hypothesis, patients with CKD experience a disruption of the “healthy” microbial community in the gut. Gut microbiota produce large quantities of PBUTs, such as IS, PCS, and TMAO, which contribute to kidney damage. Increased gut barrier permeability allows these PBUTs to enter the bloodstream. Toxins produced by harmful bacteria accumulate in the blood, causing persistent inflammation, oxidative stress, immune responses, and alterations in microbial metabolism and composition. This forms a vicious cycle, leading to the development and progression of CKD and its complications (29, 30). In a large-scale clinical cohort study involving 223 patients with ESRD and 69 gender- and age-matched healthy controls, metabolomics analysis indicated that gut microbiota in patients with ESRD caused metabolic alterations characterized by the accumulation of several PBUTs and secondary bile acids (31). Furthermore, our team conducted deep metagenomic sequencing on different stages of kidney disease compared to healthy individuals, identifying 54 high-quality microbial genome-assembled genomes with differential presence. Functional analysis revealed more genes encoding PBUTs, antibiotic resistance, and virulence factors in functional groups positively correlated with disease severity, suggesting a role of PBUTs in the occurrence and progression of kidney disease (32).

Accumulated PBUTs in patients with CKD inhibit the expression of genes associated with tight junction proteins such as zonula occludens-1 and claudins, leading to intestinal barrier damage and increased permeability (33–35). This allows PBUTs to enter the bloodstream and damage the kidneys. IS and PCS can increase reactive oxygen species (ROS) content and triphosphopyridine nucleotide (NADPH) oxidase activity in a time-dependent manner, significantly raising downstream mRNA expression of transforming growth factor-β (TGF-β1) and tissue inhibitor of metalloprotease-1 (TIMP-1), thereby damaging renal tubular cells (36, 37). Additionally, IS and PCS activate the local renin-angiotensin system in the kidneys, increasing levels of TGF-β1, promoting sustained renal hyperperfusion and fibrosis (38–40). Various antigens produced by potential pathogens in the gut can activate immunity, triggering a cascade of inflammatory reactions, leading to a unique state of “chronic inflammatory immune suppression” that induces glomerulosclerosis and tubulointerstitial fibrosis (41–43). Renal biopsy results show significant aggregation of M1 macrophages in early CKD stages, transitioning to M2 macrophages in later stages, promoting renal damage repair and fibrosis (44–46). In CKD mouse models, antibiotic treatment can reduce M1 and M2 polarization in bone marrow-derived macrophages induced by TMAO, thus alleviating renal fibrosis progression (47).Studies indicate that PCS interferes with antigen presentation by macrophages and inhibits T helper cell 1immune responses, leading to adaptive immune dysfunction in patients with CKD (48). PCS also directly inhibits macrophage immune responses and reduces peripheral blood B cell counts (29).

In addition to causing common oxidative stress, inflammation, and immune effects that contribute to renal damage in patients with CKD, PBUTs can also alter autophagy and epigenetic states, furthering CKD progression. A clinical study found almost non-existent mRNA levels of autophagy-related genes in patients with ESRD, indicating impaired autophagy activation in patients with CKD (49). DNA methylation, an important epigenetic regulatory mechanism, requires methyl donors derived from choline metabolized by gut microbiota. In the absence of gut microbiota, intestinal DNA methylation levels significantly decrease (50–52).

### Cardiovascular effects of PBUTs

An increasing number of studies have linked the aforementioned PBUTs with cardiovascular mortality in patients with CKD. Compared to non-CKD patients, those with CKD exhibit a twofold higher CVD mortality rate, with the risk increasing with the severity of CKD (53). In a study involving 147 patients with CKD, elevated plasma IS levels were associated with major adverse cardiovascular events, independent of glomerular filtration rate (GFR) and nutritional status (54). Clinically, elevated plasma TMAO levels have been established as an independent predictor of cardiovascular risk and validated in a large prospective cohort study (55). Two meta-analyses indicated that higher TMAO levels are associated with a 23%-67% increased risk of CVD events and a 55%-91% increased risk of all-cause mortality (56, 57). Additionally, in a cohort of 4000 coronary artery disease patients undergoing selective diagnostic cardiac assessment, those with higher plasma levels of PAGln, especially patients with concomitant T2DM, experienced higher rates of major adverse cardiac events over three years (23, 58).

The impact of PBUTs on cardiovascular pathology primarily manifests as arteriosclerosis, thrombosis, vascular calcification, neointimal hyperplasia, and myocardial fibrosis, ultimately leading to conditions such as myocardial infarction, heart failure, arrhythmias, stroke, and peripheral artery disease. TMAO promotes thrombosis and exacerbates arteriosclerosis through mechanisms involving macrophage scavenger receptors, foam cell activation, endothelial cell activation, increased platelet reactivity, and inhibition of reverse cholesterol transport (59, 60). Additionally, TMAO induces tissue factor and vascular cell adhesion molecule-1 expression in human microvascular endothelial cells, significantly enhances Ca^2+^ stimulation and platelet aggregation, influences vascular calcification, and promotes the progression of arteriosclerosis (61). Long-term dietary supplementation of carnitine in mice alters gut microbiota composition, significantly increasing the synthesis of TMA and TMAO, which aggravates arteriosclerosis. However, inhibiting gut microbiota prevents this phenomenon (62). Thus, modifying gut microbiota to reduce the production of protein-bound uremic toxins can improve cardiovascular outcomes.

IS, a classic PBUT, enhances platelet activity, increases reactions involving collagen and thrombin, and elevates microparticles and platelet-monocyte aggregates derived from platelets. IS binds to the ligand-binding domain of AhR in endothelial cells and vascular smooth muscle cells, activating AhR and inducing tissue factor transcription (63, 64). Concurrently, IS induces ROS expression and stimulates the expression of inflammatory cytokine, mediating thrombosis, vascular injury, and myocardial fibrosis (65, 66).

### Interaction between PBUTs and metabolic syndrome

A meta-analysis of 25 cohort studies, 3 cross-sectional studies, and 19 case-control studies confirmed that obesity increases the risk of developing CKD in the general population (67). Elsa et al. (68) demonstrated that overweight and obesity in middle-aged individuals increase the risk of CVD by 31% and 76%, respectively, while in older adults, the risks are elevated by 22% and 40%, respectively. Additionally, approximately 20%-40% of diabetic patients also have kidney disease (69), with CVD being a major cause of morbidity and mortality in patients with T2DM (9). In a large retrospective study involving over ten thousand participants with an average age of 41.8 years and a follow-up period of 3.7 years, the presence of metabolic syndrome was analyzed for its association with all-cause mortality and CVD mortality risk. The results indicated that metabolic syndrome increases the risk of all-cause mortality in women and CVD mortality risk across the entire population (70). This increased risk of metabolic disorders is primarily caused by insulin resistance and obesity, ultimately leading to chronic dysfunction of the heart and kidneys, highlighting the need for early intervention and aggressive treatment upon diagnosis of metabolic syndrome.

#### PBUTs and obesity

An increasing number of studies indicate that disruptions in gut microbiota and the accumulation of PBUTs play significant roles in the development of obesity. For instance, individuals with severe obesity exhibit higher proportions of *Firmicutes* and *Proteobacteria* compared to healthy obese and lean individuals. These bacteria can metabolize dietary components into precursors of uremic toxins such as phenylacetic acid (PAA) and TMA (71, 72). A meta-analysis exploring the association between circulating TMAO levels and obesity risk reveals a positive correlation between TMAO and increased BMI (73). Furthermore, a dose-dependent relationship between TMAO and obesity has been observed even in seemingly healthy individuals. Using 16S rRNA sequencing and untargeted metabolomics to analyze differences in gut microbiota, plasma, and intestinal metabolism between rats fed a high-fat diet and those on a normal diet, it was found that changes in gut microbiota included decreased abundance at the phylum level and reduced levels of *Akkermansia*, *Ralstonia*, *Bacteroides*, and *Faecalibacterium* at the genus level; Significant alterations were also observed in intestinal and plasma metabolite levels (74). Previous studies have demonstrated that Sangzhi alkaloids (SZ-A) alleviate high-fat diet-induced obesity, improve fat tissue metabolism, and reduce inflammation associated with obesity (75). Oral administration of SZ-A significantly reduces body weight, fat mass, total cholesterol, and low-density lipoprotein levels in high-fat diet-induced obese mice. Interestingly, SZ-A also modulates gut microbiota and alters fecal metabolite composition in obese mice. Compared to the high-fat diet group, SZ-A improves the proportions of *Firmicutes* and *Proteobacteria* at the phylum level and significantly increases the abundance of *Bacteroidetes* and *Akkermansia muciniphila* at the genus level. This change affects the relative abundance of microbial genes involved in PBUTs metabolism. Overall, SZ-A alleviates obesity and metabolic syndrome in high-fat diet-induced obese mice by improving gut microbiota and their metabolic characteristics (76).

#### PBUTs and diabetes

Research has confirmed a link between alterations in gut microbiota and the accumulation of PBUTs with host insulin sensitivity, glucose metabolism, and impaired amino acid metabolism related to DM. Our team previously identified changes in gut microbiota in patients with DM (77). In a case-control study involving thousands of newly diagnosed T2DM cases and controls, plasma TMAO concentrations were measured and found to be elevated in patients with DM (78). Within patients with DM, gut microbiota convert tryptophan into indole and its derivatives, which act on the AhR pathway, leading to reduced production of glucagon-like peptide-1 (GLP-1) and Interleukin-22. This contributes to increased intestinal permeability and translocation of lipopolysaccharides, resulting in inflammation, insulin resistance, and hepatic steatosis (79). Screening of 130 patients with T2DM revealed that IS levels increase with urinary proteinuria, and PCS also showed an upward trend in urine (80). Treatment of non-DM and early-stage hyperglycemic diabetic mice with a sodium-glucose co-transporter-2 inhibitor for one week not only lowered blood glucose levels but also reduced the formation of PBUTs such as IS by gut microbiota, thereby decreasing their systemic exposure and the need for renal detoxification. This provides a metabolic basis for kidney and cardiovascular protection (81, 82). Therefore, as blood glucose levels rise in patients with DM, exacerbating renal excretory burden, accumulated PBUTs play a “bridging” role, manifesting as a triangular relationship among “diabetes, gut microbiota/PBUTs, diabetic complications,” demonstrating interactions, mutual influences, and mutual development among these three entities.

## Nutritional intervention

### Low-protein diet

Dietary intervention has long been the cornerstone of treatment for patients with CKM syndrome, aimed at reducing the intake of precursors to PBUTs. This approach addresses the root cause to diminish PBUTs production, thereby preventing the onset and delaying the progression of CKM syndrome.

The 2022 edition of *the CKD Early Screening, Diagnosis, and Treatment Guidelines* (82) specifies protein intake recommendations for both non-diabetic CKD and diabetic CKD patients, emphasizing a reduction in overall protein intake while ensuring adequate high-quality protein intake. An experimental evaluation of a low-protein diet (LPD) among CKD patients demonstrated significant reductions in serum levels of IS and PCS in those adhering to LPD (83). Additionally, the PREDICT 1 study from the UK, involving thousands of participants, correlated gut microbiota composition with habitual diets and cardiovascular metabolic markers in blood. The research identified specific components of the microbiota associated with dietary intake and multiple measures of cardiovascular metabolic health, suggesting the potential use of gut microbiota as biomarkers for cardiovascular metabolic risk and strategies to reshape the microbiota to improve personalized dietary health (84). Among healthy species, *Firmicutes* showed the highest correlation; while *Clostridium* difficile was associated with overall poor health. These gut microbiota all participated in the metabolic processes of PBUTs. In addition to a LPD with adequate high-quality protein, CKM syndrome patients, especially those in stages II-IV of CKD, often suffer from complications such as calcium and phosphorus metabolism disorders, hyperkalemia, and hypertension. Therefore, they also need to adhere to low-phosphorus, low-potassium, and low-sodium diets.

## Special dietary patterns

### Mediterranean diet

The Mediterranean diet (MD) is characterized by high consumption of vegetables, legumes, fruits, nuts, whole grains, and dairy, abundant use of extra virgin olive oil, and encourages the selection of lean proteins (85). It has protective effects against CKD syndrome, obesity, diabetes, chronic kidney disease, and cardiovascular diseases (86–89). A meta-analysis of 70 studies analyzed the correlation between adherence to the MD and the risk of major chronic diseases (T2DM, CKD, and CVD), consistently demonstrating a significant negative association between higher adherence to the MD and the risk of these chronic diseases (86). Adherence to the MD pattern is associated with distinct characteristics of the gut microbiota.

An evaluation of the gut microbiota and metabolome of 153 individuals with different dietary habits, stratified by diet type and adherence to the MD, revealed a significant correlation between vegetable-based diets and increased levels of fecal short-chain fatty acids(SCFAs), *Prevotella*, and certain fiber-degrading *Firmicutes.* Conversely, higher urinary TMAO levels were detected in individuals with lower adherence to the MD (90). An 8-week isocaloric MD dietary intervention study (n=82) showed changes in various microbial features in the gut, including increased abundance of major dietary fiber metabolites, decreased PBUTs metabolites, and beneficial changes in cardiovascular metabolic biomarkers (90).

Additionally, a parallel randomized controlled trial involving 82 healthy overweight and obese participants found significant reductions in plasma cholesterol in the MD group compared to the control group consuming a regular diet. Metagenomic analysis indicated changes in the gut microbiota reflecting increased gene richness in participants with reduced systemic inflammation and PBUTs during the intervention. Higher levels of microbial carbohydrate degradation genes associated with fiber degradation and butyrate metabolism were also observed (91).

### Intermittent fasting

Clinical trials on overweight adults have shown that intermittent fasting is beneficial in various contexts such as obesity, diabetes, and cardiovascular diseases (92, 93). Intermittent fasting is increasingly recognized as a promising approach to managing CKM syndrome, potentially improving lifestyle and cardiovascular metabolism to prevent the onset of T2DM and CVD. Su et al. (94) evaluated the impact of intermittent fasting on gut microbiota in young and middle-aged healthy non-obese individuals, finding significant reshaping of the gut microbiota with increased *Clostridiaceae*, *Helicobacteraceae*, and butyrate-producing bacteria, and decreased *Prevotellaceae*, which produce TMAO. These changes contributed positively to blood sugar levels, weight, and body fat. However, a follow-up study involving thousands of diabetic patients found a linear inverse correlation between daily eating frequency and overall mortality as well as cardiovascular disease-related mortality, with HRs (95% CIs) of 0.88 (0.80-0.98) and 0.77 (0.63-0.93), respectively (95). Another large-scale follow-up experiment over 8.0 years found that intermittent fasting was significantly associated with increased cardiovascular mortality risk (95). Currently, there is no consistent conclusion on whether intermittent fasting is beneficial in clinical conditions. Evidence suggests that higher eating frequency is associated with a lower risk of metabolic syndrome and hypertension (96, 97), while another study indicates that higher eating frequency is associated with blood pressure in adults without cardiovascular disease and diabetes, as well as the progression rate of new-onset hypertension (98). Therefore, larger studies and standardized experimental protocols are needed to determine the association between eating frequency and mortality rates. This could potentially serve as a strategy for daily dietary management in diabetic patients and offer valuable insights into preventing premature mortality from CKM syndrome.

## Exercise intervention

In the preliminary management of CKM syndrome, priority should be given to addressing the impact of adverse social determinants of health and improving patients’ unhealthy lifestyle habits such as diet and exercise (6). Exercise is a cost-effective lifestyle intervention that can prevent and treat obesity, T2DM, and their complications, and is closely linked to microbiota research (99, 100). A study randomized 39 pre-diabetic patients who had not previously received drug treatment into a sedentary control group and an exercise training group. Significant reductions in weight and obesity were observed across the entire exercise group; Improvements were also noted in insulin sensitivity, lipid profiles, cardiorespiratory health, and levels of adipose factors associated with insulin sensitivity. Further metagenomic sequencing identified significant changes in the relative abundance of *Firmicutes, Bacteroidetes*, and *Proteobacteria*. To understand how exercise-induced changes in gut microbiota regulate host metabolism, KEGG enrichment analysis indicated increased gene abundance in the sedentary group involved in producing phenolic derivatives (indole and p-cresol) and sulfates from aromatic and sulfur-containing amino acids. Subsequently, fecal microbiota from both groups were transplanted into mice, revealing similar trends in body composition, oxygen consumption, and respiratory exchange rates among mice transplanted with feces from exercise participants. Mice receiving fecal transplants from exercise participants showed significant decreases in glucose and insulin levels, marked improvements in glucose handling, and exhibited a microbial metabolite profile distinct from that observed in humans (101). Similarly, another study transplanting feces from exercise participants into mice revealed that metabolites produced by the microbiota could modulate the gut-brain axis to regulate exercise motivation (102).

## Strategies for managing gut microbiota

### Probiotics

The use of probiotics may help improve the progressive changes in CKM syndrome (103, 104). Probiotics can colonize the human intestinal tract, enhance gut microbiota, regulate metabolism, and maintain intestinal balance. Clinical studies have revealed the positive effects of probiotic interventions on glucose metabolism, particularly the hypoglycemic effects of *lactobacilli* and *bifidobacteria*, which have been confirmed in multiple clinical trials (105, 106). Akbari et al. (107) found that probiotic supplementation significantly reduces insulin resistance, fasting blood glucose, and glycosylated hemoglobin levels. In a clinical study recruiting 38 patients with CKD who used specific probiotic capsules daily for 6 months, the average estimated glomerular filtration rate (eGFR) decline rate significantly slowed from an average monthly decline of 0.54 (-0.18 to -0.91) to 0.00 mL/min/1.73 m² (+0.48 to -0.36), and serum levels of inflammatory cytokine and IS were significantly reduced following probiotic use (108). Additionally, a 6-week randomized, placebo-controlled, crossover trial, administering heat-killed *Lactococcus*, *Lactobacillus acidophilus*, and *Bifidobacterium longum* to patients with CKD reduced plasma PCS levels, alleviating cardiovascular damage caused by PBUTs accumulated in CKD. Some preliminary evidence suggests that probiotics may protect cardiovascular metabolism in CKM syndrome (109). For instance, a randomized controlled trial reported beneficial effects of Brewer’s yeast in heart failure patients, improving left ventricular ejection fraction (110). However, probiotics administered to critically ill patients with weakened immunity may become opportunistic pathogens causing endocarditis (111), indicating that careful consideration is needed before administering probiotics to vulnerable groups.

### Prebiotics

“Prebiotics” refers to substrates selectively utilized by host microorganisms that confer health benefits (112). Prebiotics naturally exist in many fruits, grains, vegetables, honey, and breast milk.

In a single-center, double-blind, placebo-controlled trial involving overweight or obese children aged 7-12 years, participants were randomly assigned to either a prebiotic or placebo group for 16 weeks. Compared to children receiving the placebo, those in the experimental group experienced significant decreases in weight score (3.1% decrease), body fat percentage (2.4% decrease), and trunk fat percentage (3.8% decrease), while the placebo group showed increases (0.5%, 0.05%, and -0.3%, respectively). Additionally, Interleukin 6 levels and triglycerides decreased significantly from baseline in the experimental group, while the placebo group showed a 25% increase. 16S rRNA sequencing indicated a significant increase in *Bifidobacterium* species in the experimental group and a decrease in ordinary pseudo-bacteria species (113). Professor Zhao Liping designed and developed a diet called WTP, consisting primarily of various whole grains, traditional Chinese medicinal food homologues, and prebiotics. Multiple clinical trials have shown that this dietary fiber can significantly enhance the ability of intestinal bacteria in obese and diabetic patients to produce SCFAs. Further studies have demonstrated that similar dietary interventions can improve gut microbiota composition and metabolic health (114).

### Fecal microbiota transplantation

Fecal microbiota transplantation (FMT) is the most direct method to reshape gut microbiota. Initially recommended for treating recurrent *Clostridioides* difficile infection (115), FMT has also shown efficacy in managing diabetes (116), obesity metabolic syndrome (101), and CKD (117). Anne Vrieze et al. (118) successfully applied FMT to patients with metabolic disorders, finding significantly increased insulin sensitivity in obese patients with metabolic syndrome after gut microbiota infusion from lean donors. In a study by Pieter de Groot’s team, 20 diabetic patients were randomly assigned to receive either autologous or allogeneic (healthy donor) FMT. Results showed significantly preserved stimulated C-peptide levels in the autologous FMT group at 12 months. A negative correlation was found between small intestinal *Prevotella* and residual β-cell function (r=-0.55, p=0.02), while plasma metabolites correlated positively with residual β-cell preservation (rho=0.56, p=0.01 and rho=0.46, p=0.042) (119). Early research from our team found significant alleviation of symptoms in diabetic complications following FMT from healthy donor fecal microbiota (120). Animal models, divided into control, CKD, and CKD+FMT groups, showed significant improvement in disrupted gut microbiota, reduced PCS accumulation, and improved glucose tolerance after FMT treatment (121).

FMT therapy is increasingly accepted and recognized as a “natural” therapeutic method for treating various diseases due to its effectiveness, safety, and convenience. It has become a hot topic of interest among biologists, clinicians, and other stakeholders and is rapidly evolving. However, FMT poses unique and complex challenges for clinicians and regulatory agencies, including unclear mechanisms of action, definitions of healthy donors, screening procedures, sample preparation, storage conditions, dosing responses, administration methods, and settings. These factors may limit its broader application in clinical practice and hinder its expansion.

## Drug therapy

The AHA has established detailed staging criteria and clinical management recommendations for CKD-MBD syndrome. These guidelines can be utilized for managing CKD-MBD syndrome at different stages, aiming to improve cardiovascular and renal outcomes. For CKD-MBD stages 0 and I, the focus is on lifestyle adjustments to maintain normal weight and other health indicators. For CKD-MBD stage II and beyond, individualized pharmacological treatment is recommended (6).

The AHA recommends considering medications with cardio-renal protective effects to improve gut microbiota and alleviate CKD syndrome outcomes. These medications include angiotensin-converting enzyme inhibitors (ACEIs), angiotensin II receptor blockers (ARBs), glucagon-like peptide-1 receptor agonists (GLP-1RAs), sodium glucose co-transporter 2 inhibitors (SGLT-2is), and novel non-steroidal mineralocorticoid receptor antagonists (MRAs) like finerenone (6, 122–125). A study involving 36 patients treated with ACEIs/ARBs and 19 untreated patients conducted 16S rRNA sequencing and fecal metabolomics analysis. It showed that ACEI/ARB treatment improved gut microbiota by reducing potentially pathogenic bacteria such as *Escherichia coli* and *Klebsiella* spp. and increasing beneficial bacteria like *Odoribacter* spp. Additionally, significant metabolic changes were associated with ACEI/ARB treatment (126). Empagliflozin, an SGLT2i, not only improves hyperglycemia but also reduces weight, lowers blood pressure, and decreases cardiovascular events and mortality (127, 128). Our research team found that the cardiovascular benefits of empagliflozin might be related to changes in gut microbiota and plasma metabolites. In a randomized, open-label, 3-month clinical trial involving 76 newly diagnosed T2DM patients with CVD risk factors, patients were administered either empagliflozin or metformin. Both groups showed significant reductions in HbA1c levels and improvements in glucose metabolism. However, only the empagliflozin group improved cardiovascular disease risk factors, significantly reshaped gut microbiota after one month, elevated levels of SCFAs-producing bacteria such as *Roseburia*, *Ruminococcus*, and *Faecalibacterium* spp., and decreased levels of harmful bacteria including *Escherichia coli-Shigella*, *Bilophila*, and *Hungatella* spp (129). To further understand the primary mechanisms of SGLT2i, non-diabetic and diabetic mice with early and mild hyperglycemia were treated with SGLT2i for one week. This treatment revealed impacts on cardiac and hepatic signaling, with more pronounced effects observed in white adipose tissue, showing increased lipolysis. These effects were particularly influenced by gut microbiota capable of fermenting phenylalanine and tryptophan into cardiovascular PBUTs, with a lower relative abundance of specific bacterial taxa (81).

Similarly, the 2023 ESC Guidelines for managing cardiovascular disease in diabetes patients emphasize the importance of comprehensive management to reduce cardiovascular and renal failure risks, recommending first-line therapies such as non-steroidal MRAs with established cardiovascular and renal benefits (130). Animal studies have shown that compared to spironolactone, finerenone improves myocardial and renal hypertrophy, reduces BNP and proteinuria levels, and decreases the expression of pro-inflammatory and pro-fibrotic genes in cardiac and renal tissues (131).The Phase 3 FIDELIO-DKD and FIGARO-DKD trials confirmed that in patients with T2DM-related CKD, finerenone not only provides renal protection but also improves cardiovascular outcomes. The FIDELITY trial (n = 13,026) further demonstrated the clinical benefits of finerenone, with a 14% reduction in composite cardiovascular risk (HR 0.86, 95% CI: 0.78, 0.95) and a 23% reduction in composite renal outcomes risk (HR 0.77, 95% CI: 0.67, 0.88) (132).

Currently, no studies link gut microbiota and its metabolites to finerenone both domestically and internationally. However, previous research has shown that steroidal mineralocorticoid receptor antagonists can alter the composition and diversity of gut microbiota in hypertensive patients, impacting intestinal barrier permeability and sympathetic nervous system function (133). Therefore, further exploration is needed to determine whether the cardiovascular and renal benefits of finerenone in CKD syndrome patients are mediated through changes in gut microbiota and its toxin-like metabolites as potential targets.

## Others

### Klotho protein

The Klotho protein is a protective transmembrane protein in the kidneys (134). Animal experiments have shown that IS and PCS can inhibit Klotho expression, activate the renin-angiotensin-aldosterone system (RAAS) and TGF-β pathways, ROS generation, exacerbate oxidative stress and inflammation, and promote renal tubulointerstitial fibrosis formation (135, 136). In a clinical study involving 86 predialysis patients, serum Klotho levels were found to decrease as eGFR declined. Additionally, the interaction between elevated IS levels and increased left ventricular mass was more pronounced in patients with low Klotho levels (137). Serum Klotho levels gradually decrease in CKD patients, while levels of the inflammatory factor Tumor Necrosis Factor-α increase. This significant correlation indicates a close association between reduced Klotho protein and the development of CKD-related microinflammatory states (138).

Studies consistently indicate that Klotho can prevent uremic toxin-related cardiac toxicity by inhibiting oxidative stress to suppress IS-induced endothelial dysfunction (138). Treatment with Klotho protein in a CKD-related left ventricular hypertrophy mouse model significantly inhibited the development of left ventricular hypertrophy (137). Additionally, Klotho protein therapy can prevent IS-induced thrombosis and atherosclerosis in apolipoprotein E knockout mice (139). Therefore, exogenous supplementation of Klotho may be a potential therapeutic approach to inhibit the progression of uremic cardiomyopathy.

### Aryl hydrocarbon receptor

Aryl hydrocarbon receptor (AhR) is a ligand-dependent transcription factor widely expressed in immune, epithelial, endothelial, and stromal cells within barrier tissues. Recent studies indicate that AhR signaling serves as a critical mediator in the progression of diseases induced by various PBUTs, contributing to intestinal homeostasis between the host and gut microbiota (140). All tryptophan metabolites—indole uremic solutes and kynurenic acid—are agonists of the AhR pathway (141, 142). Cardiovascular disease is a leading cause of mortality associated with CKD-MBD syndrome. In a rat model of cardiac hypertrophy, hypertension, and myocardial fibrosis induced by 5/6 nephrectomy, AhR pathway activation was observed, including AhR translocation and downstream protein Cytochrome P450 1(CYP1) expression, accompanied by increased ROS production detected via staining. Experimental evidence demonstrated that IS triggers AhR translocation, leading to significantly increased downstream gene expression, and that AhR inhibitors, CYP1 inhibitors, and AhR-targeting siRNA effectively block ROS production. Moreover, inhibition of the AhR/CYP1/ROS pathway collectively attenuates IS-mediated cardiomyopathy promotion. This research highlights the activation of the AhR/CYP1 pathway in disease, specifically associated with the uremic toxin IS (143). The central role of AhR in the progression of CKD-MBD syndrome and its activation by PBUTs provide compelling evidence supporting AhR as a therapeutic target in the later stages of CKD-MBD. Resveratrol, a natural AhR antagonist, suppresses proteinuria, hypoalbuminemia, and hyperlipidemia in nephritic rats (144). Addi et al. (145) confirmed that CH223191, a specific AhR antagonist, reduces the expression of TF in human endothelial cells during the PBUTs indole-3-acetic acid-mediated process. Meanwhile, Assefa et al. (146) demonstrated that resveratrol attenuates vascular endothelial activation and permeability by blocking the IS/AhR pathway, thereby exerting cardiovascular protective effects.

Despite AhR’s potential as a promising clinical target, much of our understanding of its physiological and pathological functions in CKD-MBD syndrome comes from animal models, complicating the translation to clinical applications. Therefore, further research is essential to unravel the complex roles of AhR, ensuring its safe and effective use in prevention and treatment.

### AST-120

AST-120 is an oral medication designed to adsorb and remove precursors of PBUTs produced in the gastrointestinal tract. It is currently the only drug known to improve PBUTs symptoms in patients with CKD and delay the need for dialysis (147). AST-120 effectively lowers circulating and renal levels of IS (148, 149). In Japan, it has been widely used in patients with CKD to clear intestinal precursors of PBUTs. Recent case studies by Tomino et al. (150) reported that patients with CKD receiving AST-120 showed an increase in GFR, with kidney function improving rapidly and progressing to ESRD upon discontinuation of treatment. Another study found that AST-120 therapy significantly reduced levels of total IS, PCS, free IS, and free PCS (151). A prospective randomized study on stage II and IV patients with CKD demonstrated that IS levels decreased in the AST-120 treatment group but not in the control group. Besides preserving kidney function and clearing toxins, a retrospective study indicated that AST-120 helped reduce the incidence of cardiovascular events and mortality rates in patients with CKD (152). Additionally, animal experiments have shown that AST-120 can prevent the progression of arteriosclerosis in a mouse model of chronic renal failure by preserving levels of anti-angiogenic factors (153).

**Figure 2:**
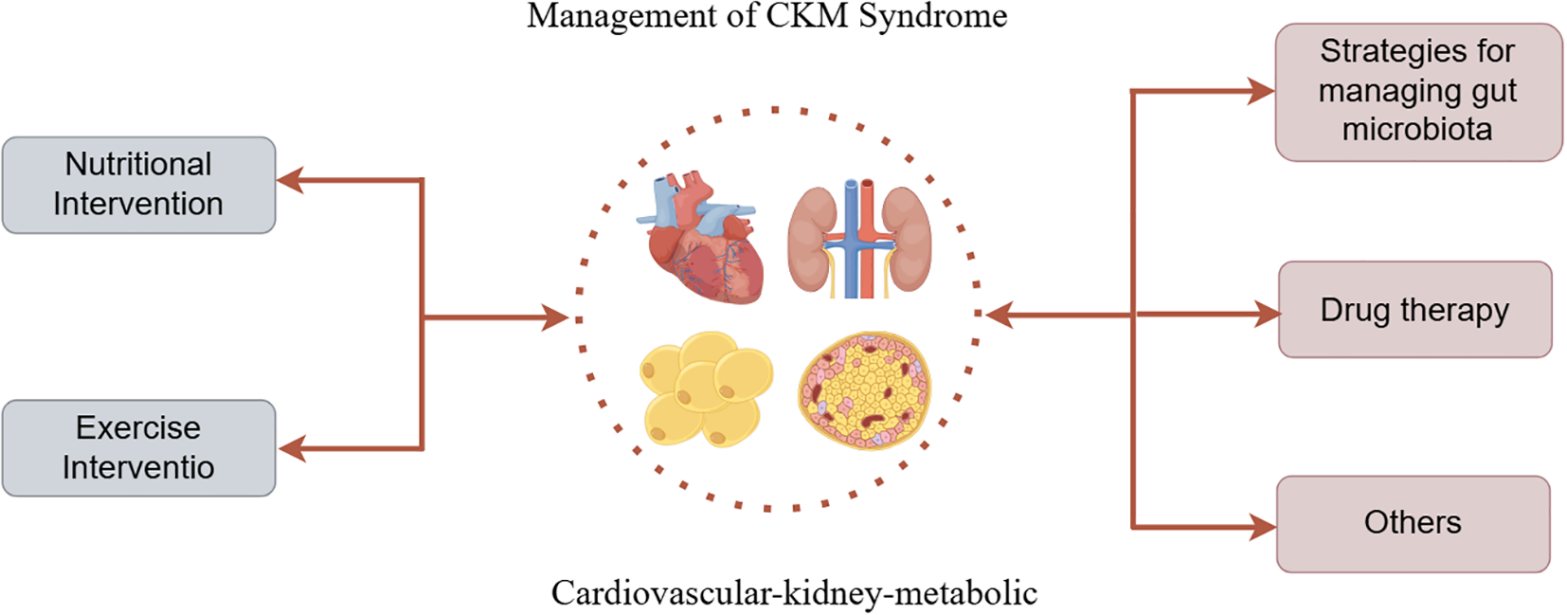
Management of CKM syndrome: targeting PBUTs.

Therefore, in addition to traditional drug treatments for CKM and related diseases, therapeutic approaches targeting the gut microbiota primarily include dietary and lifestyle interventions, supplementation with beneficial bacteria, FMT, and research into new drugs. Both supplementation with beneficial bacteria and FMT essentially involve the introduction of exogenous probiotics. Existing studies have shown that they can alleviate disease by increasing the abundance of beneficial gut bacteria, competing for limited nutrients to inhibit the growth of pathogenic bacteria, and reducing the production of PBUTs by pathogenic bacteria. However, its application is limited due to the difficulty of supplementing probiotics in establishing long-term colonization in the host and the risk of transmission of viruses and diseases from donors. The clinical effect of traditional drug treatment in CKM is not good and the progression of the disease cannot be controlled, so there is a greater need for innovative treatments to reduce PBUTs to maintain the overall state of the disease, but at present, a large number of clinical experimental studies and validations are needed. In conclusion, the treatment of PBUTs needs to be developed urgently (**Figure 2**).

## Prospect

The AHA report emphasizes that CKM syndrome is a systemic and progressive pathophysiological process. It is not merely an aggregation of several diseases, but rather a result of the mutual influences and promotion among metabolic diseases such as diabetes, CKD, and CVD. As the disease progresses, the accumulation of PBUTs generated through metabolism exacerbates these conditions, ultimately leading to cardiorenal damage. Therefore, early detection and prevention are crucial in treating CKM syndrome. Clinical practitioners need a clear understanding of CKM syndrome and should actively screen at-risk populations. Developing new screening and risk prediction models for CKM syndrome will help prevent CVD events associated with it, significantly delaying its progression and improving patient survival rates. Our preliminary cross-sectional study found distinct gut microbiota characteristics in healthy individuals, early-stage patients with DKD, and late-stage patients with DKD, with variations in core genome content associated with toxin production. We identified 54 core genomes capable of significantly distinguishing patients with DKD from healthy individuals and demonstrating good discriminatory ability among patients with DKD of different severities (32). Therefore, gut microbiota and PBUTs hold promise as a CKM syndrome screening and risk prediction model, addressing early screening challenges and enabling proactive prevention and treatment to avoid adverse cardiorenal outcomes.

PBUTs, characteristic of the CKM syndrome environment, have been the focus of numerous experimental and clinical approaches aimed at mitigating their pathogenic effects and halting the progression of cardiorenal outcomes. However, further discussion is needed to evaluate whether these methods can reverse the CKM syndrome state and their impact on prognosis. Additionally, detailed staging of CKM syndrome requires the adoption of multiple modalities to adjust clinical management across different stages, facilitating precise, targeted therapy against these toxins.

The introduction of the novel concept of CKM syndrome represents not only a redefinition of the disease status but also a comprehensive update of treatment paradigms. Addressing PBUTs in the treatment of CKM syndrome offers a promising approach to overcoming challenges in screening, predicting, and managing the progression of CKM syndrome. This approach aims for the integrated, holistic management of patients with CKM syndrome.
